# An Outbreak of Porcine Reproductive and Respiratory Syndrome Virus (PRRSV) in a German Boar Stud: A Retrospective Analysis of PRRSV Shedding in Boar Semen

**DOI:** 10.3390/vetsci11110557

**Published:** 2024-11-11

**Authors:** Jakob Aundrup, Caroline Lüken, Kristin Heenemann, Thomas W. Vahlenkamp, Isabel Hennig-Pauka

**Affiliations:** 1 Swine Health Service, LUFA Nord-West, Institute for Animal Health, Ammerländer Heerstraße 123, D-26129 Oldenburg, Germany; 2 Veterinary Practice Melle, Lammersbrink 8, D-49328 Melle, Germany; 3 LUFA Nord-West, Institute for Animal Health, Ammerländer Heerstraße 123, D-26129 Oldenburg, Germany; 4 Centre for Infectious Diseases, Institute of Virology, Faculty of Veterinary Medicine, University of Leipzig, An den Tierkliniken 29, D-04103 Leipzig, Germany; 5 Field Station for Epidemiology, University of Veterinary Medicine Hannover, Foundation, Buescheler Str. 9, D-49456 Vechta, Germany

**Keywords:** PRRSV, semen, boar stud, PRRSV shedding

## Abstract

The porcine reproductive and respiratory syndrome virus (PRRSV) causes high economical costs due to reduced productivity and losses in pig production. The virus can infect sow herds through various routes. One possible risk factor is the transmission of PRRSV through artificial insemination with infected boar semen. For these reasons, conventional boar studs should be closely monitored to detect an outbreak of PRRSV at an early stage. In the presented retrospective study, 2184 fresh semen samples from 336 boars were investigated (RT-qPCR) after an accidental PRRSV introduction to the herd. Hence, the different shedding profiles of PRRSV via semen resulted in 42.2% where no virus was detected, 2.0% intermittent shedding, and 8.4% permanent shedding. The duration of viral shedding varies from 2 days to 83 days post outbreak (on average 33 days). A significant impact of breed on PRRSV shedding duration could not be shown. Also, the distribution of the shedding profile does not follow a consistent mode, indicating that not every boar is shedding the virus via semen.

## Simple Summary

In this retrospective and longitudinal field study, PRRSV was detected in fresh boar semen using PCR. The aim of the study was to reconstruct the duration and shedding profiles of PRRSV in semen using field data. The detection of PRRSV in semen proved to be difficult due to low virus concentrations and the presence of inhibitors that affect PCR accuracy. Shedding patterns were highly variable, with some boars shedding the virus intermittently, continuously, or not at all over a period of weeks to months. Regular monitoring of the boars included testing of serum and semen samples, with PRRSV often detected earlier in blood than in semen. The study demonstrates the importance of combining PCR and antibody testing to improve detection accuracy and timing. In addition, the results suggest that enhanced biosecurity measures such as air purification systems and strict hygiene protocols are crucial for controlling the spread of PRRSV in boar studs. Comprehensive surveillance and early detection are necessary to minimize PRRSV transmission, maintain boar health, and prevent further outbreaks.

## 1. Introduction

The porcine reproductive and respiratory syndrome virus (PRRSV) is one of the most relevant viral diseases in swine production worldwide [1,2]. It causes major problems in pig health and leads to losses in productivity. There are only a few countries that are free of PRRSV in their swine population (e.g., Switzerland, Sweden, Norway, Finland, and Australia) [3,4,5,6]. Pigs of all ages are generally susceptible. However, respiratory disease primarily affects suckling piglets, weaners, and fattening pigs, whereby the reproductive form of the disease mostly affects sows varying from poor conception rate, late term abortions, weak live-born and stillborn piglets [1,2]. Generally, affected adult pigs do not show severe clinical signs [7]. In sows, the presence of manifest reproductive disorders can be one of the first indications of PRRSV infection. In boars, a variable reduction in semen quality can be observed [8].

PRRSV belongs to the Arteriviridae family [9]. It is a small, enveloped, single-stranded RNA virus classified into two species with PRRSV-1 being predominant in Europe (EU-type) and PRRSV-2 being predominant in America and Asia (US-type). The virus replicates primarily in monocyte-derived cells (e.g., alveolar macrophages) and its high mutation rates has resulted in an unique genetic and antigenic diversity [10]. In boars the virus can be found in reproductive tissues [11]. Viremia can occur 24 h post infection and can be present for approximately 2 weeks in adult pigs [7]. In experimentally infected boars, seroconversion could be observed between 11 and 14 days post inoculation with PRRSV [12].

Virus transmission can be divided into a direct and an indirect route [2]. The indirect route includes transmission via vectors, e.g., equipment, instruments, staff, and aerosols [2]. In regions with a high pig density, aerosol transmission is considered by some authors to play a crucial role [13]. Aerosol transmission depends on various climatic factors (e.g., air humidity, temperature, and solar radiation) [13]. For the direct transmission, nasal, oral, vaginal, and intrauterine routes of exposure are described [2].

In this context, routes of shedding are crucial from an epidemiological point of view. In general, infected pigs shed the virus via nasal and oral secretions, urine, feces, and semen, whereas the level and duration of virus excretion vary individually [2]. Shedding of PRRSV in semen is of particular relevance for transmission due to the long distances the virus can overcome by this route until sows are infected during artificial insemination [14]. The risk of transmission by this route depends on the concentration of the virus in the semen [2]. The standard dilution during preparation of the ejaculates should, therefore, contribute to a reduction in the risk. Nevertheless, the transmission of PRRSV during insemination with infected ejaculates has been demonstrated in several cases [14,15,16]. Efforts will, therefore, continue to keep boar studs free of PRRSV, as also stipulated since 2021 in COMMISSION REGULATION (EU) 2020/686.

Several case reports described an outbreak of PRRSV in a naïve boar stud [17,18]. Because adult boars show mostly no or less clinical symptoms after PRRSV infection [7,19], regular monitoring by blood samples (PCR and ELISA) for PRRSV is crucial, and various monitoring schemes exist [20,21]. In addition, semen samples can be tested for PRRSV themselves. It has to be taken in mind, that PRRSV is detected during the acute phase of infection in 51% of pigs in serum and least frequently in semen (22%) in the same animals [18].

The duration of virus shedding via semen varies and can be up to 92 days after experimental infection [22,23], regardless of viremia or of the presence of neutralizing antibodies [24,25]. Furthermore, the breed seems to have an impact on the duration of PRRSV shedding [22]. It is of high importance that infected boars do not always shed the virus via semen and, on the other hand, that the detection of PRRSV in semen by PCR is of lower sensitivity than with blood [7,22]. Several experimental studies on the shedding of PRRSV via semen have been performed [11,19,25], but field observations with larger sample sizes are rare [18].

In this retrospective and longitudinal field study PRRSV was detected by PCR in fresh boar semen. The duration and profile of PRRSV shedding in semen were reconstructed by field data.

## 2. Case Presentation

**Figure 1:**
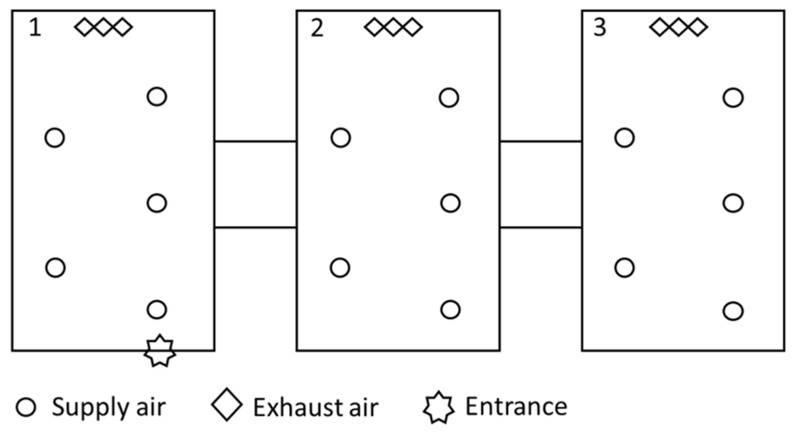
Boar stud. Unit 1, 2 and 3.

The boar stud is located in northwestern Germany and divided into three different units. Entrance is only possible via a sanitary sluice in front of unit 1. For staff and visitors, showering is mandatory before entering and protective in-house clothing is obligatory. For external visitors, a 48 h pig-free period is prescribed. The connecting covered outdoor passageways prevented contact with the outside environment. The boar stud is equipped with a slight negative pressure ventilation system. Incoming air is channelled inside the unit by five supply tubes as shown in Figure 1.

During the PRRSV outbreak, 354 boars of different breeds were housed in total (Table 1). In this study, the day of the first PRRSV detection in serum (day 0) was defined as the start of the outbreak (0 DPO). All data refer to the day post outbreak (DPO) (e.g., age and duration of PRRSV shedding). In the routine monitoring at the defined DPO, 5 out of 15 serum samples were positive for PRRSV in the RT-qPCR while serological results were negative in the ELISA. Two days later, at the follow-up examination, there were 20 boars in unit 1 and 2 were PRRSV positive in the ELISA. This means that DPO, as a definition of the outbreak start, is artificial and may have been earlier (up to 8 days) because the first PRRSV-positive results can be expected 8 days after virus contact [26].

### 2.1. PRRSV Monitoring

Before PRRSV introduction, the stud was considered PRRSV unsuspicious, according to the guideline of the swine health service [20]. The regular monitoring for PRRSV was carried out every two weeks by real-time reverse transcription polymerase chain reaction (RT-qPCR) and enzyme-linked immunosorbent assay (ELISA) of the serum [20]. Regularly, between 15 and 20 boars were sampled. Additionally, semen was examined on the alternate weeks by using RT-qPCR. Samples were analyzed in pools of five.

### 2.2. Outbreak of PRRSV and Initial Diagnostic Measures

During the routine monitoring, 5 out of 15 boars tested positive for PRRSV (Ct-values; range: 28.1–31.9) in blood serum (RT-qPCR) in unit 3 on DPO 0. ELISA results were negative (range: −0.001–0.059 (S/*p* value)). Immediately, unit 3 was separated from the other two units to prevent the virus from spreading to the other boars in unit 1 and 2. A separate entrance to unit 3 was established and only the staff responsible for this unit was allowed to enter. Contact boars in neighbouring pens in unit 3 (*n*= 10) and randomly selected boars (*n* = 10) from units 1 and 2 were sampled immediately. Contact boars in the same unit 3 tested positive by RT-qPCR (4/10, 40%) and by antibodies (4/10), 0.429–0.646 (S/*p* value). Most fresh semen samples were tested positive by RT-qPCR (7/11; Ct-values; range: 27.3–38.3) from unit 3. Boars in the neighbouring units had already become infected with PRRSV (RT-qPCR in blood 3/10 positive).

During further routine semen collection in the boar stud, every fresh semen sample was tested for PRRSV prior to further processing (RT-qPCR). In case of positive results, the respective boar was blocked for further semen collection for at least three weeks. Next, semen testing was performed after three weeks. The samples were not collected according to a strict protocol.

### 2.3. Laboratory Diagnostical Methods

Virus RNA was extracted from serum samples with a MagMAX™ Pathogen RNA/DNA Kit (ThermoFisher Scientific Inc., Waltham, MA, USA) or with a MagMAX™ CORE Nucleic Acid Purification Kit (ThermoFisher Scientific Inc., Waltham, MA, USA). RNA extraction from semen was performed with a QIAamp^®^ Viral RNA-Kit and RNeasy Mini Kit (Qiagen, Hilden, Germany). Positive PCR products were subsequently purified using the GeneJET™ Gel Extraction Kit (ThermoFisher Scientific Inc., Waltham, MA, USA).

The extracted RNA was examined for PRRSV-specific RNA using the multiplex RT-qPCR virotype^®^ PRRSV RT-PCR Kit (INDICAL BIOSCIENCE, Leipzig, Germany), according to the manufacturer’s instructions, allowing differentiation of PRRSV-1 and -2 and highly pathogenic PRRSV-2. To obtain a more detailed characterization of the circulating PRRSV strain, a PRRSV-open reading frame (ORF) 2–7 and a PRRSV-ORF 5 and ORF 7 RT-PCR was performed [27,28,29,30]. Sequencing was carried out using the dideoxy method according to Sanger (Microsynth AG, Balgach, Switzerland). The resulting nucleotide sequence was subsequently analyzed using GENtle (Magnus Manske, Cologne, University of Cologne, Germany), and alignment and comparison with sequences in the database was performed using the Basic Local Alignment Search Tool of the National Center for Biotechnology Information (NCBI; https://blast.ncbi.nlm.nih.gov/Blast.cgi [accessed on 21 February 2023]). Phylogenetic analyses and construction of phylogenetic trees were carried out by using the software MEGA X [31].

PRRSV antibodies in serum were analyzed by ELISA (HerdCheck^®^ PRRS X3 Antibody Test Kit Idexx, Ludwigsburg, Germany) according to the manufacturer’s specifications.

### 2.4. Data Collection, Measurements and Analyses

Data were assessed retrospectively considering diagnostic results from the defined first outbreak day (day of first virus detection = 0 DPO) until day 83 DPO. A database of boars including breed, age, and number of tests (RT-qPCR) was established. In total, 18 boars selected due to anomalies (e.g., poor semen quality) were not included in the evaluation even though they had been tested by RT-qPCR. These boars were prematurely culled. For evaluation of the shedding period, only boars from which at least four semen samples had been tested for PRRSV were considered. The duration of shedding represented the period from the day of introduction (DPO 0) to the last point of PRRSV detection in the semen of each individual boar, taking into account that there was always a three-week interval between semen testing. Descriptive analyses of data are presented as numbers and percentages for categorial variables and means, medians and, standard deviations for continuous variables. The metric data were tested for normal distribution. The Kruskal–Wallis test was used for statistical differences between the breeds and PRRSV shedding in semen.

## 3. Results

### 3.1. Testing of Semen Samples

**Figure 2:**
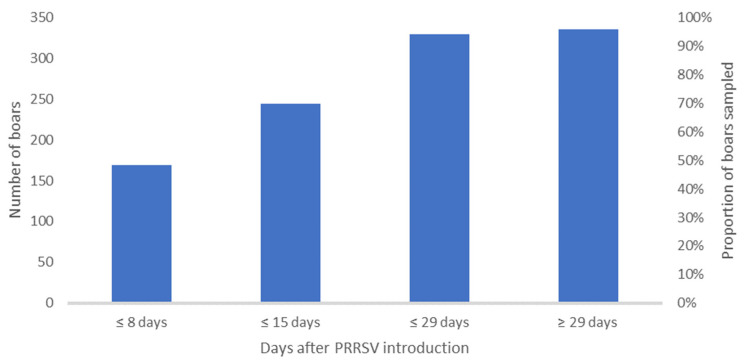
Cumulative number and proportion of boars selected for their first semen examination after the virus entry at 0 DPO in the boar stud.

A total of 2184 fresh semen samples from 336 boars were evaluated. This is an average of 6.5 tests per boar, with a range of 1 to 17 semen tests for PRRSV by RT-qPCR per boar (Table 2). In total, 390 of these samples were positive for PRRSV (17.9%). The PCR product of one PRRSV-ORF 2–7 RT-PCR was purified and sequenced. The sequence was deposited in NCBIGen Bank (NCBI accession no.: PP785695.1). The NCBI Blast analysis revealed the highest identity of 87.83% in 3587 base pairs with a PRRSV field strain with the NCBI acc. no.: FJ349261.1. Comparison with the vaccine strains currently applied in the field resulted in the highest identity in ORF 7 with 92.95% on a nucleotide basis (nt) and ORF 2b 94.29% on an amino acid basis (aa) (Supplementary Materials, Table S1). Additionally, further sample material was analyzed using PRRSV ORF 2–7 specific RT-PCR. Unfortunately, the viral load in the samples was too low to generate an additional PCR product. Therefore, PRRSV-ORF 5 and ORF 7 RT-PCRs were performed. In both RT-PCRs, the PCR products from the additional samples showed 100% identity with the sequence described in NCBI (PP785695.1). In addition, the phylogenetic tree of the partial sequences of PRRSV is shown in Supplementary Materials, Figure S1. After 15 days, at least one semen sample was obtained from 245 boars (73%). Until 29 days, 98 % of the boars (330 boars) had been tested (Figure 2).

### 3.2. Profile of PRRSV Shedding in Semen

Based on semen testing results for PRRSV of the individual boars over time, four profiles were defined:

1. Shedding of PRRSV (a): virus detection at least in one semen sample;
2. Permanent (p): PRRSV was found in at least the last two semen samples without PRRSV-negative samples between (maximum until 83 DPO);
3. No shedding (n): no virus detected in fresh semen samples;
4. Intermittent shedding (i): intermittent detection of PRRSV in a minimum of four samples but with negative samples in between.

**Figure 3:**
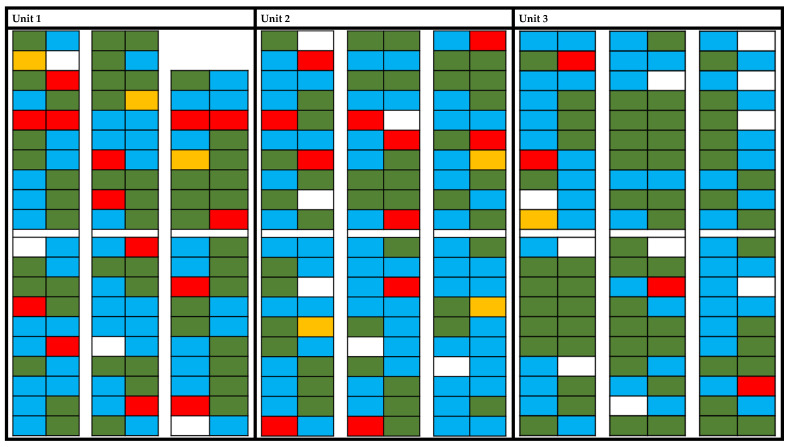
Distribution of the semen shedding profiles in the boar stud by individual boars in their respective pens. Green: no shedding (n); blue: shedding of PRRSV (a); red: permanent (p); yellow: intermittent shedding (i). White not considered.

In Table 3, the different profiles of PRRSV shedding in semen are shown separated by unit, and in Table 3, the numbers of tests are presented. In 144 boars, no virus could be detected by RT-qPCR in semen (42.8%), while in 192 (57.2%) at least one testing was positive for PRRSV; of these, 28 boars (8.4%) have shed PRRSV in the semen by at least inin the last two time points of examination (permanent). In Figure 3 the distribution of the shedding profiles located in the 3 different barns is shown.

Table 4 shows the different shedding profiles of the individual breeds. In total, 10.7% of the Pietrain boars show a permanent shedding profile, while the Duroc and Tempo boars show no permanent shedding profile.

For assessment of the duration of shedding, 375 positive semen samples were considered, irrespective if boars were classified as profile a, i, or p (Table 5). The days between first positive and last positive sampling were recorded. Positive boars show a shedding period of a minimum of 2 DPO until 83 DPO. The average period of shedding is 35 DPO. No significant difference in semen shedding between different breeds was found (*p* > 0.05)

## 4. Discussion

From an epidemiological point of view, transmission of PRRSV by means of PRRSV-positive semen through artificial insemination is a relevant risk factor. It is crucial to detect PRRSV introduction into boar studs at an early stage to prevent transmission of the virus via semen and, thus, several monitoring protocols have been elaborated. These schedules are always a compromise between cost- and time-efforts and sensitivity [15,16] arious methods for sampling and testing for PRRSV in boar studs with their respective pros and cons are performed [22,24,32,33,34]. In the present boar studs, regular monitoring for PRRSV was carried out on the serum every two weeks (RT-qPCR and ELISA) [20]. Additionally, fresh semen was examined on alternate weeks by RT-qPCR. PRRSV detection in semen by RT-PCR is challenging [7,35]. In semen, PRRSV might exist in low concentrations, making it difficult to detect using conventional RT-PCR methods. This necessitates sensitive detection techniques and optimized protocols to amplify and detect even low levels of viral RNA [12,22,35]. Furthermore, semen contains various components that may inhibit the RT-PCR reaction or interfere with the detection of viral RNA [36].

In the present case, it was demonstrated that in serum samples the RT-qPCR was positive for PRRSV in 5 out of 15 at 0 DPO, while serological results were negative. Contact boars in unit 3 and randomly selected boars from units 1 and 2 (*n* = 20) were detected to be positive already two days after first detection of PRRSV. These serum samples were already positive for PRRSV antibodies suggesting that these animals had been earlier infected and that the 0 DPO defined in this study is not the real one, but instead artificial. Furthermore, a large proportion of fresh semen samples was already positive for PRRSV on 2 DPO. Pepin et al. (2015) conducted an experimental study that focused on the detection patterns of PRRSV in blood, saliva, and semen following vaccination with PRRSV-2 Modified Live Virus (MLV). PRRSV RNA was detected as early as day 1 post infection in blood, while antibodies were detectable no earlier than 9 and 10 days after infection. First PRRSV-positive semen samples were identified at the earliest 3 days after infection [37]. Comparing these results with the current case study, it becomes clear that PRRSV introduction into the boar station was detected relatively late, which is a known risk for most monitoring programmes established in practice. Generally, PRRSV monitoring protocols should always be based on PRRSV RT-qPCR (serum and semen) in combination with antibody testing (ELISA) [20,32,37]. This is also supported by a case report of a PRRSV outbreak in a Danish boar stud, where boars were only tested for antibodies (ELISA) prior to the outbreak. After the outbreak, the monitoring protocol was changed to RT-qPCR and ELISA [38].

Rovira et al. (2007) evaluated different surveillance protocols for detecting PRRSV in boar studs. In the protocols based on RT-PCR of serum, PRRSV introduction was detected earlier than in the protocols based on PCR in semen and also earlier than in protocols based on serological findings (ELISA). The most intensively evaluated protocol (testing 60 boars three times a week using RT-PCR in serum) would take 13 days to detect 95% of PRRSV introductions. These results confirm the field observations and suggest that an intensive surveillance protocol must be implemented on a boar stud to detect PRRSV introduction on an early stage [32].

Several prospective studies aimed to understand the dynamics of PRRSV shedding in semen with respect to duration and pattern of shedding by testing for PRRSV RNA in semen from infected boars [12,24,32,33,34,37]. The analyses of factors influencing the likelihood and intensity of virus shedding in semen revealed an extended period of shedding of 92 days post infection on average [23], ranging from a few days to several months. These differences were assumed to be dependent on various factors as the virus strain, amount of virus, and individual host factors leading to variation among boars [7]. Studies have shown that PRRSV shedding in semen can occur during the acute phase of infection and can persist even in the absence of clinical signs [2,11,19,39]. Shedding typically begins within a few days to a week after infection and can continue intermittently or permanently for an extended period [33,34]. In our study, the detection of PRRSV in fresh semen was observed for the first time on two DPO, the average detection period was 35 days, and the maximum detection period extended up to 83 DPO. Due to financial restrictions, testing frequency was limited because it is known that a positive boar stayed positive for some weeks with a high probability. Due to the fact that some animals already seroconverted, the correct time point of entry could have been approximately 14 days earlier, which is the average duration of viraemia, which finally depends on the virus strain. The results of our study are consistent with a previous study by Christopher et al. 1995. In that study, the experimentally infected boars shed PRRSV via semen up to 92 days post inoculation (dpi), while it was detected in the bulbourethral gland until 101 dpi [23].

In case of a positive PRRSV fresh semen sample, the boar was blocked for a minimum of three weeks. During this time, no information about virus shedding was gathered. It cannot be stated whether the permanent shedding definition includes recurrent shedding, because no semen had been sampled within the three-week period. It cannot be stated if the non-shedders would have been detected as shedders if semen samples would have been examined within the three-week intervals. The definition of profiles is an artificial classification to report the observations in a standardized way. There is no information available regarding how long PRRSV persists or survives in semen/accessory glands in the case that no semen is collected. There is no information regarding if viral RNA of a dead virus will be eliminated within three weeks, so that testing will show a negative result. The specific mechanisms of how PRRSV infects the reproductive tract and whether it replicates independently are not fully understood [7,11].

Various PRRSV shedding patterns have been described as some infected boars shed the virus permanently, while others exhibit intermittent shedding or become non-shedders [23]. In the presented case, in 144 boars no virus could be detected in semen (42.8%), while in 55.8% at least one testing was positive for PRRSV. Of these, 28 boars (8.1%) shedded PRRSV permanently. Also, in the distribution of PRRSV shedding profiles (Figure 2), no pattern according to time of infection or location within the unit could be identified as, e.g., also neighbouring boars show different shedding profiles.

It is known that the immune response to PRRSV in an individual is influenced by various factors such as genetic background, age, and health status [2]. Duroc boars and Tempo boars excreted PRRSV in their semen on for a shorter time on average Pietrain or 410 boars, which excreted the virus for an average of 37 days. This observed difference was not significant (*p* > 0.05). It cannot be excluded that the time of PRRSV entry into the stud can also lead to different shedding profiles. Boars might have been in different stages of infection due to the fact that only some animals had seroconverted and were viraemic. Different virus strains have different abilities to infect and replicate in individual pigs [40]. In the case that more than one PRRSV strain entered the herd, neighbouring boars could have been infected with different PRRSV strains leading to the observed differences in shedding profiles. Sequencing data were generated for one positive sample in ORF 2 to 7 and for other samples in ORF 5 and ORF 7 with an identity of 100%. So, the presence of different strains can be excluded. Unfortunately, it was not possible to determine the exact introduction route of the PRRSV into the boar stud. The probability that it was transmitted via the air was considered to be high.

So far, efforts were ongoing to eradicate PRRSV from the boar stud. Vaccination with an inactivated vaccine or modified live vaccine (MLV) was discussed to reduce virus shedding via semen. An MLV was found to be successful in reducing the shedding of PRRSV-2 in boar semen following an experimental challenge [41]. In this case, it was decided that the boars would not be vaccinated. Rapid spontaneous infection with PRRSV was confirmed in the boar stud by weekly blood samples examined for viraemia in parallel to the semen samples and results indicated high transmission rates of the virus. Finally, it was found that all boars were infected with PRRSV rapidly. After 83 days, the 144 non-shedder boars (N) were tested for PRRSV in serum. The RT-qPCR was negative and the ELISA tests resulted in seroconversion in 99% of the boars. The boars that permanently shed the virus (P) were slaughtered and no new boars were housed. The number of boars was reduced to such an extent that unit 3 became free. After the unit had been completely cleaned and disinfected, seronegative boars from units 1 and 2 were stalled into unit 3. After one year, the first naive boars were moved into unit 1. In addition, an air purification system was installed in the boar stud to reduce the risk of airborne transmission of PRRSV. The supply air is first channelled through filter pockets, and before the outside air enters the stud, it is purified using UV light. Unfortunately, a further PRRSV outbreak occurred during the construction phase of the supply air purification system, meaning that it was not possible to make any further statements on the remediation of the first virus outbreak.

## 5. Conclusions

This study reveals the complexity of PRRSV infection and shedding dynamics within a boar stud, which finally resulted in various shedding profiles. Implementing comprehensive biosecurity measures, regular monitoring, and appropriate management strategies are crucial to minimize the risk of PRRSV entry and transmission and maintain herd health.

## Figures and Tables

**Table 1:** General information about the boar stud.

Unit	Pens in Unit	Number of Boars in Individual Pens	Breed (410/DU/PI/TN)	Age ^1^ (Days)
1	116	116	6/17/81/12	660 (226;2059)
2	120	120	3/15/94/8	720 (223;2528)
3	120	118	8/8/89/13	710 (228;2356)
total	356	354	17/40/264/33	703.5 (223;2528)

410 = crossbreed of PIC 408 and PIC 337 boar. DU: Duroc; PI: Pietrain; TN: Large White breed. ^1^ average age of boars: Median (Min;Max).

**Table 2:** RT-PCR tests per shedding profile.

Profile	Boars/Number of Tests	Tests per Boar ^1^
A	157/900	5.73 (1;17)
P	28/166	5.93 (3;9)
N	144/1067	7.41 (2;14)
I	7/51	7.29 (5;9)
total	336/2184	6.50 (1;17)

^1^ Average (min;max).

**Table 3:** PRRSV semen shedding profiles in each unit.

Unit	Profile of PRRSV Shedding in Semen			Total
	A (Shedder)	P (Permanent)	N (No)	I (Intermittent)
1	48 (42.9)	13 (11.6)	48 (42.8)	3 (2.7)
2	62 (54.4)	11 (9.7)	38 (33.3)	3 (2.6)
3	47 (42.8)	4 (3.6)	58 (52.7)	1 (0.9)
	157 (46.8)	28 (8.4)	144 (42.8)	7 (2.0)

The number of boars and percentage is presented.

**Table 4:** Semen shedding profile of PRRSV in different breed.

Breed	Profile of PRRSV Shedding in Semen				Total
	A	P	N	I	
PI	120 (47.6)	27 (10.7)	99 (39.3)	6 (2.4)	252
Du	18 (51.4)	0	17 (48.6)	0	35
410	6 (35.3)	1 (5.9)	10 (58.8)	0	17
TN	13 (40.5)	0	18 (56.2)	1 (0,3)	32
total	157 (46.7)	28 (8.3)	144 (44.9)	7 (2.1)	336

The number of boars and the percentage is presented. 410 = crossbreed of PIC408 and PIC337 boar. DU: Duroc; PI: Pietrain; TN: large White breed.

**Table 5:** Duration of shedding of positive boars.

Breed	Number of Boars	Estimation of Duration of Shedding (Days ^1^)
PI	146	37 (2;83)
Du	12	30 (9;65)
410	6	37 (16;71)
TN	14	20 (8;49)
Total	178	35 (2;83)

^1^ Days are presented in median (min;max). 410 = crossbreed of PIC 408 and PIC 337 boar. DU: Duroc; PI: Pietrain; TN: large White breed.

## Data Availability

Data are contained within the article and supplementary materials.

## Supplementary Materials

The following supporting information can be downloaded at: https://www.mdpi.com/article/10.3390/vetsci11110557/s1, Table S1: Comparison of the ORF2 to ORF7 sequence (NCBI-Acc. No.: PP785695) with the available PRRSV vaccines strains. Figure S1: Phylogenetic tree of partial sequences.
