# Supplementary material for: An Outbreak of Porcine Reproductive and Respiratory Syndrome Virus (PRRSV) in a German Boar Stud: A Retrospective Analysis of PRRSV Shedding in Boar Semen
Source: Vet Sci. 2024 Nov 11;11(11):557. doi: 10.3390/vetsci11110557 (PMC11599107; doi:10.3390/vetsci11110557)
Supplement: Supplementary file 1 [file vetsci-11-00557-s001.zip › vetsci-3224206-supplementary.pdf]

Table S1. Comparison of the ORF2 to ORF7 sequence (NCBI-Acc. No.: PP785695) with the available PRRSV vaccines strains

	ORF 2a		ORF 2b		ORF 3		ORF 4		ORF 5		ORF 6		ORF 7	
PRRSV Stamm	% nt	% aa	% nt	% aa	% nt	% aa	% nt	% aa	% nt	% aa	% nt	% aa	% nt	% aa
Ingelvac®PRRS MLV strain 94881 [KT988004]	86.82	85.14	89.67	94.29	85.11	83.77	84.42	83.61	85.53	84.58	88.31	93.06	91.43	87.40
Suvaxyn PRRS MLV strain 96V198 [MK876228]	86.29	87.04	89.67	92.86	86.13	86.40	84.00	80.11	84.49	85.07	88.12	90.75	91.38	91.34
UNISTRAIN PRRSV strain VP-046bis [GU067771]	86.68	86.64	92.49	94.29	84.84	82.89	83.15	81.97	85.83	85.57	89.66	93.06	92.41	90.55
Porcilis® PRRS strain DV [KJ127878]	87.21	87.45	91.55	94.29	85.26	84.65	84.81	79.78	86.47	86.07	90.23	94.22	92.95	89.76

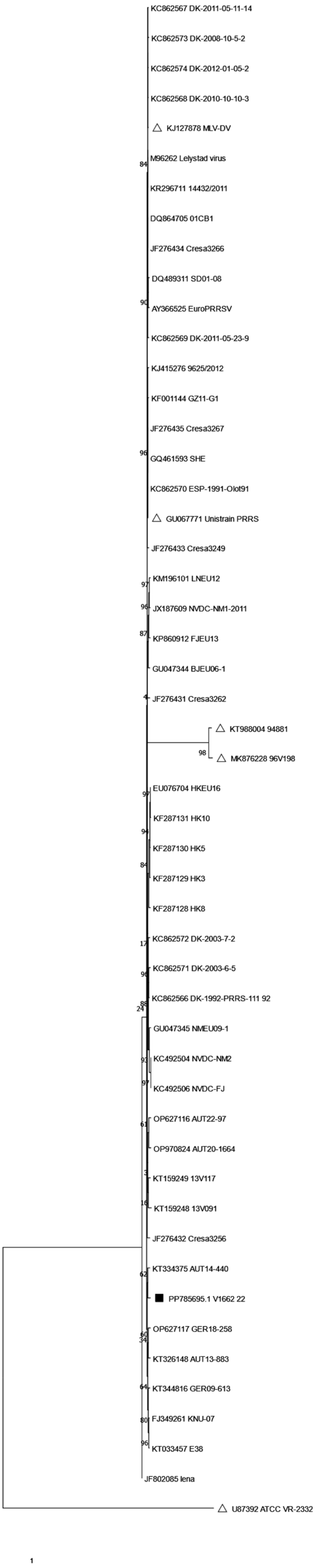
KC862567 DK-2011-05-11-14
KC862573 DK-2008-10-5-2
KC862574 DK-2012-01-05-2
KC862568 DK-2010-10-10-3
KJ127878 MLV-DV
M96262 Lelystad virus
KR296711 14432/2011
DQ864705 01CB1
JF276434 Cresa3266
DQ489311 SD01-08
AY366525 EuroPRRSV
KC862569 DK-2011-05-23-9
KJ415276 9625/2012
KF001144 GZ11-G1
JF276435 Cresa3267
GQ461593 SHE
KC862570 ESP-1991-Olot91
GU067771 Unistrain PRRS
JF276433 Cresa3249
KM196101 LNEU12
JX187609 NVDC-NM1-2011
KP860912 FJEU13
GU047344 BJEU06-1
JF276431 Cresa3262
KT988004 94881
MK876228 96V198
EU076704 HKEU16
KF287131 HK10
KF287130 HK5
KF287129 HK3
KF287128 HK8
KC862572 DK-2003-7-2
KC862571 DK-2003-6-5
KC862566 DK-1992-PRRS-111 92
GU047345 NMEU09-1
KC492504 NVDC-NM2
KC492506 NVDC-FJ
OP627116 AUT22-97
OP970824 AUT20-1664
KT159249 13V117
KT159248 13V091
JF276432 Cresa3256
KT334375 AUT14-440
PP785695.1 V1662 22
OP627117 GER18-258
KT326148 AUT13-883
KT344816 GER09-613
FJ349261 KNU-07
KT033457 E38
JF802085 lena
U87392 ATCC VR-2332
1

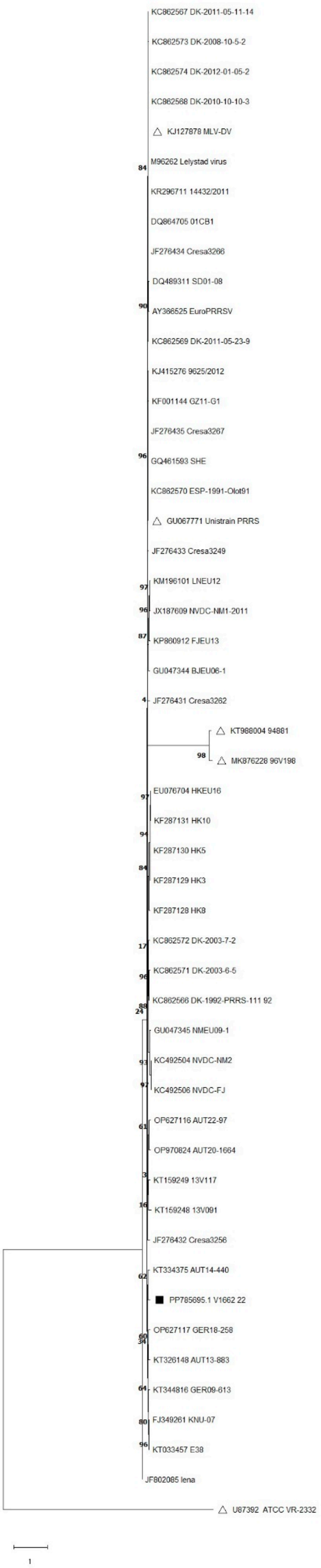
KC862567 DK-2011-05-11-14
KC862573 DK-2008-10-5-2
KC862574 DK-2012-01-05-2
KC862588 DK-2010-10-10-3
KJ127878 MLV-DV
M96262 Lelystad virus
KR296711 14432/2011
DQ864705 01CB1
JF276434 Cresa3266
DQ489311 SD01-08
AY366525 EuroPRRSV
KC862569 DK-2011-05-23-9
KJ415276 9625/2012
KF001144 GZ11-G1
JF276435 Cresa3267
GQ461593 SHE
KC862570 ESP-1991-Olot91
GU067771 Unistrain PRRS
JF276433 Cresa3249
KM196101 LNEU12
JX187609 NVDC-NM1-2011
KP860912 FJEU13
GU047344 BJEU06-1
JF276431 Cresa3262
KT988004 94881
MK876228 96V198
EU076704 HKEU16
KF287131 HK10
KF287130 HK5
KF287129 HK3
KF287128 HK8
KC862572 DK-2003-7-2
KC862571 DK-2003-6-5
KC862566 DK-1992-PRRS-111 92
GU047345 NMEU09-1
KC492504 NVDC-NM2
KC492506 NVDC-FJ
OP627116 AUT22-97
OP970824 AUT20-1664
KT159249 13V117
KT159248 13V091
JF276432 Cresa3256
KT334375 AUT14-440
PP785695.1 V1662 22
OP627117 GER18-258
KT326148 AUT13-883
KT344816 GER09-613
FJ349261 KNU-07
KT033457 E38
JF802085 lena
U87392 ATCC VR-2332
1

Prototype U87392 ATCC VR-2332 was used as the outgroup.

Figure S1. A Phylogenetic tree of partial sequences (3807 bp) of Betaarterivirus suid 1 and 2 with the virus amplified in this study (NCBI Acc.-Nr.: PP785695, labelled with a black square) and 50 additional sequences from the GenBank (vaccine strains labelled with an empty triangle). The evolutionary history was inferred by using the Maximum Likelihood method and Kimura 2-parameter model [A]. The tree with the highest log likelihood (--52583.46) is shown. Evolutionary analyses were conducted in MEGA X.
